# Modular organization across changing task demands in healthy and poststroke gait

**DOI:** 10.14814/phy2.12055

**Published:** 2014-06-24

**Authors:** Rebecca L. Routson, Steven A. Kautz, Richard R. Neptune

**Affiliations:** 1 Department of Mechanical Engineering, The University of Texas at Austin, Austin, Texas; 2 Ralph H. Johnson VA Medical Center, Charleston, South Carolina; 3 Department of Health Sciences and Research, Medical University of South Carolina, Charleston, South Carolina

**Keywords:** Biomechanics, hemiparesis, muscle synergies, walking

## Abstract

Our goal was to link impaired module patterns to mobility task performance in persons poststroke. Kinematic, kinetic, and electromyography (EMG) data were collected from 27 poststroke subjects and from 17 healthy control subjects. Each subject walked on a treadmill at their self‐selected walking speed in addition to a randomized block design of four steady‐state mobility capability tasks: walking at maximum speed, and walking at self‐selected speed with maximum cadence, maximum step length, and maximum step height. The number of modules required to account for >90% of the variability accounted for the EMG patterns of each muscle was found using nonnegative matrix factorization. Module compositions of each module during each task were compared to the average module in self‐selected walking using Pearson's correlations. Additionally, to compare module timing, the percentage of integrated module activation timing within six regions of the gait cycle was calculated. Statistical analyses were used to compare the correlations and integrated timing across tasks. Mobility performance measures of task capability were speed change, cadence change, step length change, and step height change. We found that although some poststroke subjects had a smaller number of modules than healthy subjects, the same underlying modules (number and composition) in each subject (both healthy and poststroke) that contribute to steady‐state walking also contribute to specific mobility capability tasks. In healthy subjects, we found that module timing, but not composition, changes when functional task demands are altered during walking. However, this adaptability in module timing, in addition to mobility capability, is limited in poststroke subjects.

## Abstract (precis)

The overall goal of this study was to begin linking impaired module patterns to mobility task performance in persons poststroke. We found that the same underlying modules (number and composition) that contribute to steady‐state walking also contribute to mobility capability tasks in healthy subjects and in subjects poststroke.

## Introduction

In healthy adults, the biomechanical subtasks of steady‐state walking (e.g., body support, forward propulsion, leg swing, and mediolateral balance control) have been shown to be generated by independent groups of coexcited muscles or modules (Neptune et al. 2009; Allen and Neptune 2012). However, individuals poststroke display poor intermuscular coordination characterized by a merging of modules that are normally independent in healthy individuals (Clark et al. 2010). A higher number of independent modules poststroke has been associated with improved performance in various clinical and biomechanical assessments of walking, including increased walking speed, improved ability to increase walking speed (range from self‐selected to fast), improved Dynamic Gait Index, and improved step length and propulsion symmetry (Bowden et al. 2010; Clark et al. 2010). Modules have also been shown to be associated with specific biomechanical functions during movement (Neptune et al. 2009; Allen and Neptune 2012), and the merging of modules interferes with the successful execution of the biomechanical functions (Allen et al. 2013). As more modules are merged, greater interference between subtasks occurs, leading to poorer walking performance. However, in a recent study, we found that improving walking ability with a clinical intervention resulted in improvements in the number and quality of modules poststroke (Routson et al. 2013).

In addition to steady‐state walking, daily lower limb mobility is comprised of many diverse motor tasks such as accelerating, stopping, turning, and avoiding obstacles. Studies investigating healthy individuals executing tasks such as kicking a ball while walking (Ivanenko et al. 2005), running (Cappellini et al. 2006), walking with induced slipping (Oliveira et al. 2012), and running with cutting maneuvers (Oliveira et al. 2013), have identified module patterns similar to those in walking. Some of these studies also revealed adaptability in module timing (Cappellini et al. 2006; Oliveira et al. 2013) or changes in the number of modules (Ivanenko et al. 2005) in response to changing task demands. A recent study hypothesized that the central nervous system adapts the existing module structure to task demands rather than introducing new modules (Oliveira et al. 2012). Because each module contributes to specific biomechanical functions in healthy walking (Neptune et al. 2009; Allen and Neptune 2012), we expect that mobility tasks that require changes in specific biomechanical functions will affect the corresponding module patterns and timings associated with that function. Thus, a lack of independent modules or a lack of ability to change the timing of a specific independent module as is commonly seen in subjects poststroke could affect a subject's ability to execute specific mobility tasks (e.g., increase step height, step length, or cadence).

Our goal was to: (1) explain mobility task performance within the context of impaired module patterns; and (2) develop a clinical assessment tool specific to poststroke mobility that directly relates impaired function to impairment of specific module patterns in order to guide therapeutic interventions. This would ultimately characterize an individual's overall mobility capability rather than typical mobility performance (i.e., what subjects can do vs. how subjects typically perform). As a first step toward this goal, we will define the underlying motor patterns that contribute to specific mobility tasks in healthy subjects (e.g., fastest comfortable walking [FC], high stepping [HS], long stepping [LS], quick stepping [QS]) in order to establish how a subject's ability to modify gait in response to specific changes in task demands is reflected in module composition and timing. We then will investigate these mobility capability tasks in subjects poststroke by comparing the module composition, module timing, and mobility capability performance of the poststroke and neurologically healthy subjects. Achieving this will facilitate the assessment of how specific deficits in mobility capability relate to motor control deficits in subjects poststroke, and thus enable clinical interventions guided by patient‐ and task‐specific mobility goals.

## Methods

### Experimental setup and procedure

Kinematic, kinetic, and electromyography (EMG) data were collected from 27 poststroke subjects (Table 1) with hemiparesis secondary to a single unilateral stroke. Subject inclusion criteria consisted of the following: free of significant lower extremity joint pain, range of motion limitations, and major sensory deficits; able to ambulate independently with an assistive device over 10 m on a level surface; walk on a daily basis in the home; with no severe perceptual or cognitive deficits; free of significant lower limb contractures; and no significant cardiovascular impairments contraindicative to walking. Data were also collected from 17 healthy control subjects (Table 1) free from neurological disease and lower limb orthopedic impairments. All participants provided written informed consent and the Institutional Review Board approved the protocol.

**Table 1.** Subject demographics. All poststroke subjects were at least 6 months post stroke.

Variable	Averages	SD	Range
Poststroke group (*n *=**27)			
Age	60.15	12.08	28–76
OG self‐select walking speed (m/s)	0.73	0.32	0.29–1.23
Berg balance score	47.70	6.79	25–55
Fugl‐Meyer assessment	22.85	6.95	9–34
Fugl‐Meyer assessment – Synergy	15.22	5.15	5–22
Sex (male/female)	18/9		
Control group (*n *=**17)			
Age	54.18	8.33	40–74
OG self‐select walking speed (m/s)	1.20	0.19	0.75–1.46
Sex (male/female)	9/8		

Each subject walked on a split‐belt instrumented treadmill (Bertec, Columbus, Ohio) at their self‐selected (SS) walking speed for 30‐sec trials in addition to a randomized block design of four steady‐state mobility capability tasks: walking at maximum speed (FC), and walking at self‐selected speed with maximum cadence (QS), maximum step length (LS), and maximum step height (HS). Practice trials were performed to ensure that subjects were comfortable with the experimental setup. To ensure that a steady‐state walking pattern was achieved for the data collection, subjects walked approximately 10 sec prior to data collection. For each of the mobility tasks, three trials were collected and the most successful (e.g., highest cadence) trial compared to the self‐selected walking trial was used for data analysis. Mobility performance measures of task capability were speed change, cadence change, step length change, and step height change, all with respect to the self‐selected walking trial.

### Data collection and processing

Reflective kinematic markers were placed on the limbs and torso using a modified Helen Hayes marker set. Marker locations were recorded at 120 Hz using a 12‐camera motion capture system (PhaseSpace, Inc., San Leandro, CA) and GRF data were sampled at 2000 Hz. Kinematic and GRF data were filtered using a fourth‐order Savitzky‐Golay (Savitzky and Golay 1964) least‐square polynomial smoothing filter and were resampled at 100 Hz.

Electromyography data were collected (Motion Lab Systems, Inc., Baton Rouge, LA) bilaterally from the tibialis anterior (TA), soleus (SO), medial gastrocnemius (MG), vastus medialis (VM), rectus femoris (RF), medial hamstrings (MH), lateral hamstrings (LH), and gluteus medius (GM) at 1000 Hz. EMG data were high‐pass filtered with a zero‐lag fourth‐order Butterworth filter (40 Hz), demeaned, rectified, and then low‐pass filtered with a zero‐lag fourth‐order Butterworth filter (4 Hz). To focus on temporal dissimilarities in EMG, the EMG for each muscle was normalized to its peak value during each trial. In addition, EMG was time normalized to 100% of the gait cycle. The number of modules required to account for >90% of the EMG variability accounted for (VAF) in each of the muscles was found using nonnegative matrix factorization as previously described in detail (Clark et al. 2010). For each subject, modules were identified for each mobility task separately and then an analysis of variance (ANOVA) was performed comparing the number of modules for *all* subjects across all conditions.

### Statistical analysis

All statistical analyses were performed using SAS statistical software (SAS Institute, Cary, NC). After the modules were calculated for each task for each subject, in order to create a direct comparison across tasks the self‐selected number of modules was used when comparing each mobility capability task to the self‐selected condition. Pearson's correlation coefficient was used to compare the composition of each module to the average module in SS walking (Oliveira et al. 2013; Routson et al. 2013). To enable a one‐to‐one comparison to control subjects, poststroke subjects with four modules were correlated with the control subjects. Modules in all other subject groups were correlated with their own group average SS walking data (e.g., poststroke subjects who had three modules were correlated with average SS walking data for the subjects with three modules). Higher correlations specify more similarity in module compositions. For each of the four groups of subjects (hemiparetic subjects with 2, 3, and 4 modules and healthy subjects) separate one‐way ANOVAs (*P* < 0.05) and post hoc *t*‐tests with Bonferroni corrections were used to compare the correlations across the mobility capability tasks.

**Figure 1.**
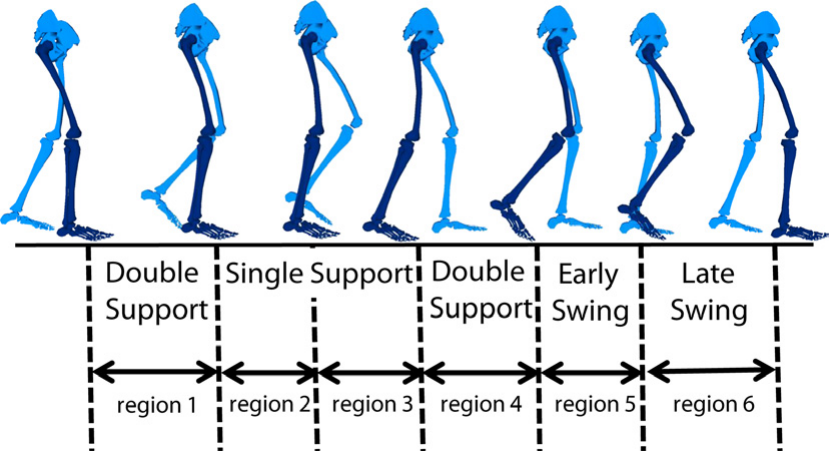
Region definitions over the gait cycle.

To assess the differences in module timing, each module's activation timing was integrated over 100% of the gait cycle and then the percentage of the total integrated module activation timing was calculated for six regions of the gait cycle (Fig. 1; Nott et al. 2014). For each of the four groups of subjects separate one‐way ANOVAs (*P* < 0.05) and post hoc *t*‐tests with Bonferroni corrections were used to compare the percentage of the total integrated module activation timing for each of the six regions of the gait cycle across the mobility capability tasks.

In addition, one‐way ANOVAs (*P* < 0.05) and post hoc *t*‐tests with Bonferroni corrections were used to compare the mobility capability performance measures (i.e., change in speed, cadence, step length, and step height) across the four subject groups.

## Results

### Control subjects

**Figure 2.**
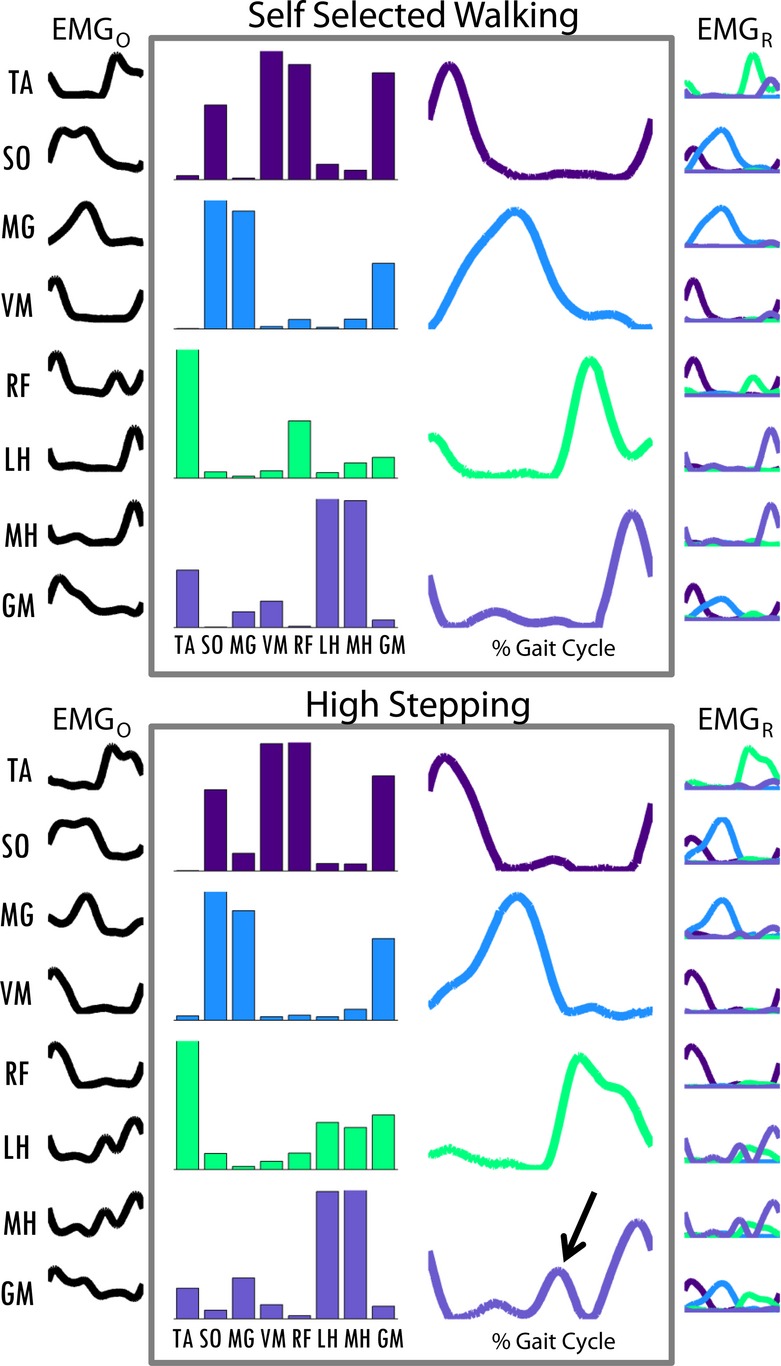
Processed electromyography (EMG; EMG_O_), module composition matrices (W, bar plots), module activation timing (C), and reconstructed EMG (EMG_R_) for a representative control subject. The top plots depict data for self‐selected (SS) walking and the bottom plots depict data for high stepping (HS) walking. The arrow points to the additional peak in Module 4 timing activation seen in HS walking. Dark purple is Module 1, blue is Module 2, green is Module 3, and light purple is Module 4. The components of each muscle's EMG_R_ due to each module are colored with the module colors, respectively.

**Figure 3.**
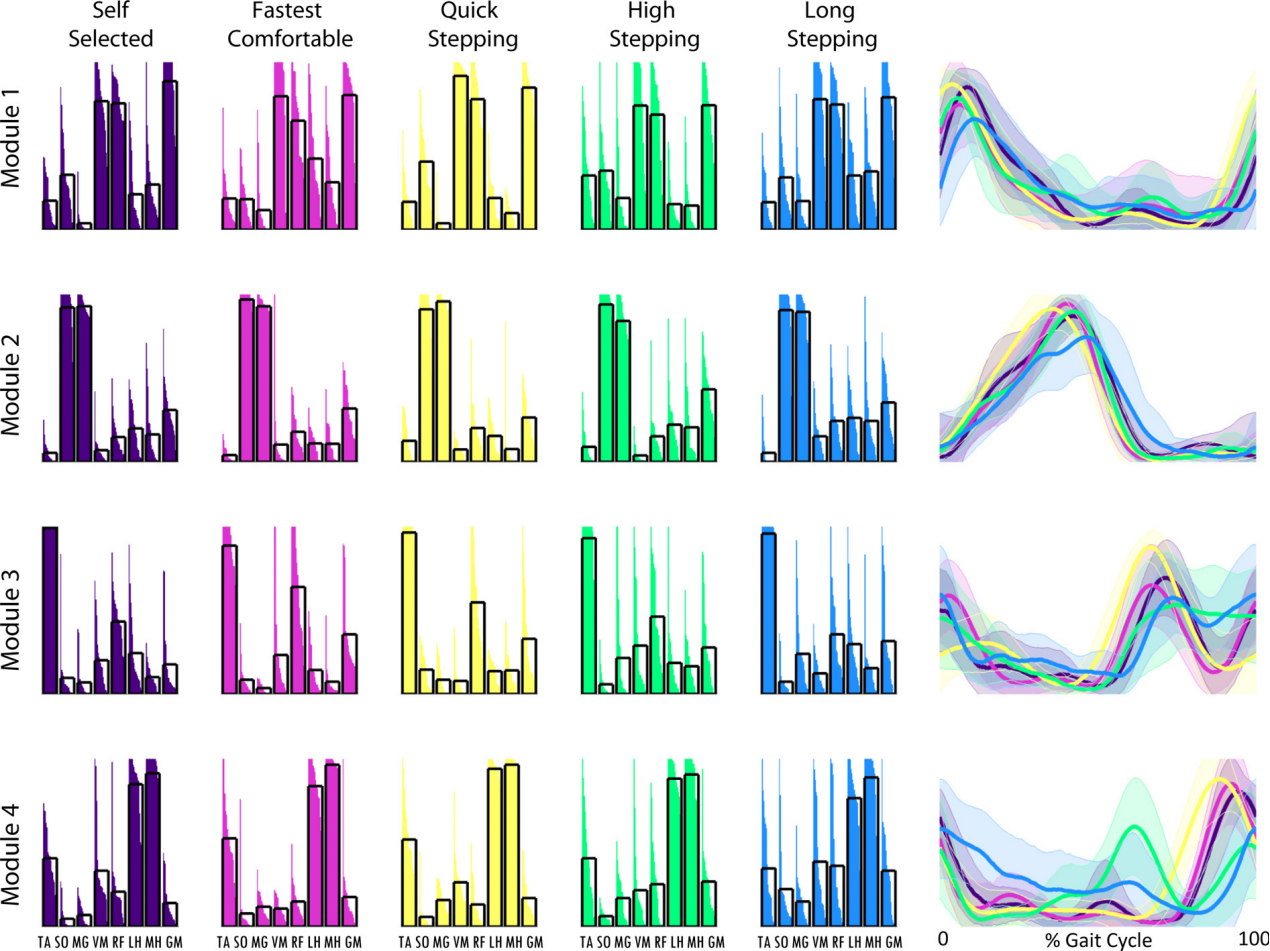
Average control subject modules for self‐selected walking (dark purple), fastest comfortable walking (pink), quick stepping (yellow), high stepping (green), and long stepping (blue). Module compositions are on the left (black boxes show average composition) and timing of the correspondingly colored module are on the right (bold lines show average and shaded areas show standard deviation). Module 1 is the top row, Module 2 is the second row, Module 3 is the third row, and Module 4 is the bottom row.

Four modules were necessary to reconstruct the EMG (e.g., Fig. 2) collected in the majority of the control subjects in all mobility tasks (3.9 ± 0.5 SS; 3.9 ± 0.4 fastest comfortable walking [FC]; 3.9 ± 0.3 quick stepping [QS]; 3.9 ± 0.6 high stepping [HS]; 3.5 ± 0.8 long stepping [LS]) with total VAF exceeding 0.98 for all tasks (0.99 ± 0.01 SS; 0.98 ± 0.01 FC; 0.98 ± 0.01 QS; 0.99 ± 0.00 HS; 0.99 ± 0.01 LS). Therefore, similar to what was previously performed to characterize healthy subject SS walking (Clark et al. 2010), typical healthy module composition and timing in all of the mobility tasks were extracted using four modules from each of the healthy subjects regardless of the number of modules assigned using the 90% of VAF criteria. In this study, the four modules observed in the control subjects for all of the mobility capability tasks were consistent with control modules previously found using the same number of modules (Fig. 3; Clark et al. 2010; Routson et al. 2013) and qualitatively similar to previous studies that recorded from a larger set of muscles (Ivanenko et al. 2004; Cappellini et al. 2006). Module 1 is composed of hip and knee extensors, Module 2 is primarily composed of the plantarflexors, Module 3 is primarily composed of the tibialis anterior and rectus femoris, and Module 4 is composed of the hamstrings.

**Figure 4.**
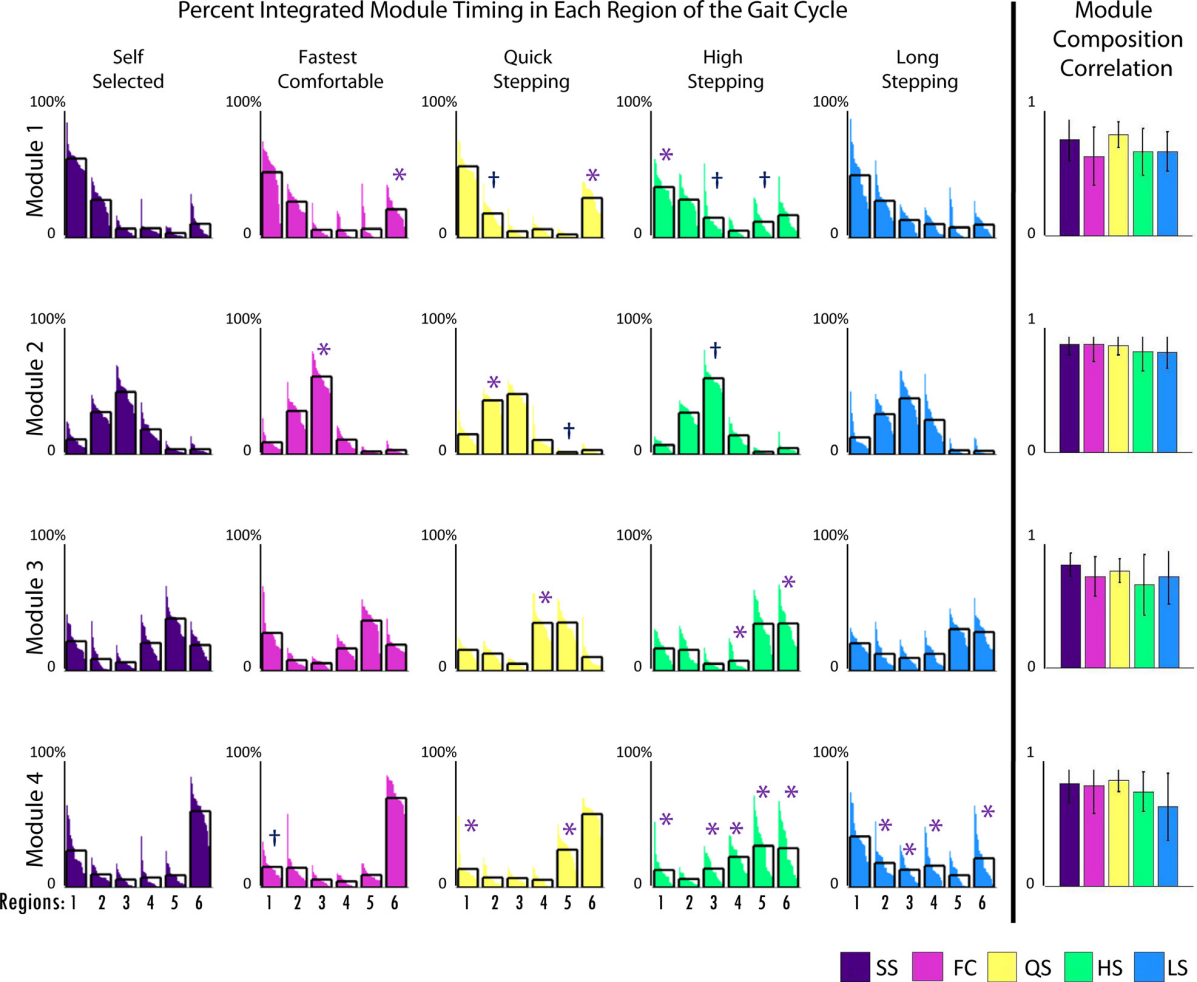
The first five bar graphs on the left represent the percent of total integrated module timing curve in each region of the gait cycle (Fig. 1) for control subjects. Analysis of variances (ANOVAs) were run for each module and region of the gait cycle across mobility capability tasks. Asterisks show significance (*α *< 0.05) and “†” shows marginal significance (*α *< 0.1) in post hoc *t*‐tests with Bonferroni corrections compared to the self‐selected (SS) condition only. The last column shows Pearson's correlations of module composition of control subjects to average module composition of control subjects. ANOVAs were run for each module across mobility capability tasks. Asterisks show significance (*α *< 0.05) in post hoc *t*‐tests with Bonferroni corrections compared to the SS condition only.

Module compositions were found to be consistent across tasks (Fig. 4). While there was a statistically significant difference in module composition in Module 4 (*P* = 0.014) in which post hoc Bonferroni *t*‐tests revealed was between LS and QS mobility tasks, this difference appears relatively minor. All of the composition correlations to the average compositions of the SS walking data were >0.65, showing a high similarity in all the module compositions during the mobility tasks to the SS condition.

However, there were clear differences in timing. The percentage of the Module 1 activation over the regions of the gait cycle (see Fig. 1) varied in FC, QS, and HS when compared to SS, with higher activation in region 6 (late swing) in FC and QS and lower activation in region 1 (early stance) in HS than in SS walking (Fig. 4). Additionally, Module 2 activation varied in mid‐stance in FC and QS when compared to SS walking with higher activation in region 3 in FC and region 2 in QS than in SS walking. Module 3 activation varied in QS and HS when compared to SS walking. In Module 3, in HS, there was a higher activation in region 6 (late swing) and lower activation in region 4 (preswing) compared with SS walking, consistent with the prolonged activation throughout swing seen in Figure 3. Also, in Module 3, there was a higher activation in region 4 (preswing) in QS compared with SS walking. The percentage of the Module 4 activation over the gait cycle varied in QS, HS, and LS when compared to SS walking. There was more Module 4 activity in region 5 (early swing) during QS than in SS walking. There was more Module 4 activity in regions 4 and 5 (preswing and early swing) and less in 1 and 6 (late swing and early stance) in HS walking than in SS walking. Also, in LS walking there was a more uniform distribution of activation of Module 4 throughout the gait cycle with higher activation in regions 2 and 4 and lower activation in region 6 than in SS walking.

### Poststroke subjects

A group analysis of *all* subjects (poststroke and control) across mobility tasks revealed no significant difference between the number of modules for any of the tasks (one‐way ANOVA; *P* = 0.78). Of the 27 poststroke subjects, 6 had two modules, 15 had three modules, and 6 had four modules in the steady‐state walking condition.

### Poststroke subjects with four modules

**Figure 5.**
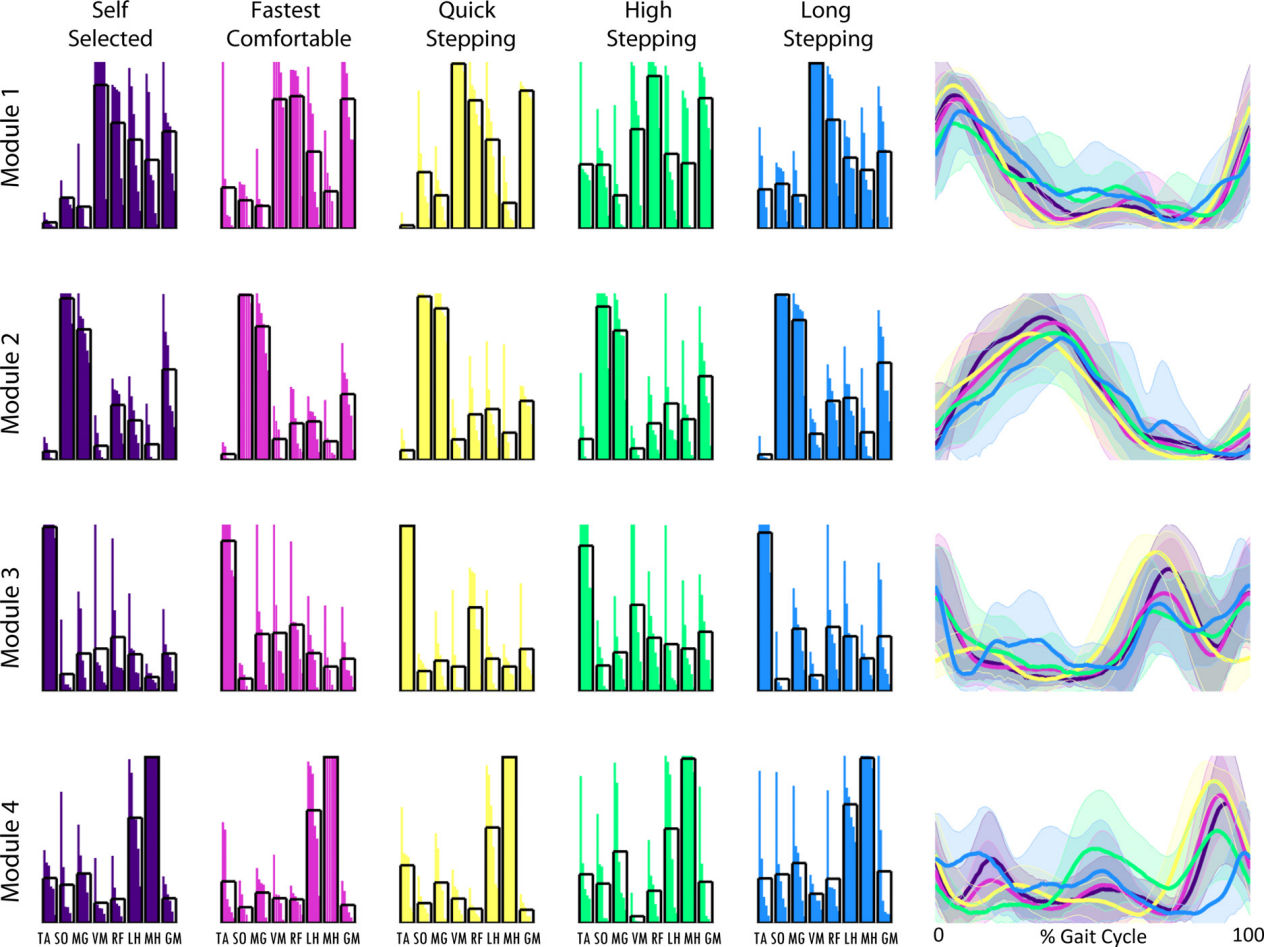
Average poststroke subject modules for subjects with four modules are shown for all tasks. Module compositions are on the left and timing of the correspondingly colored module are on the right.

**Figure 6.**
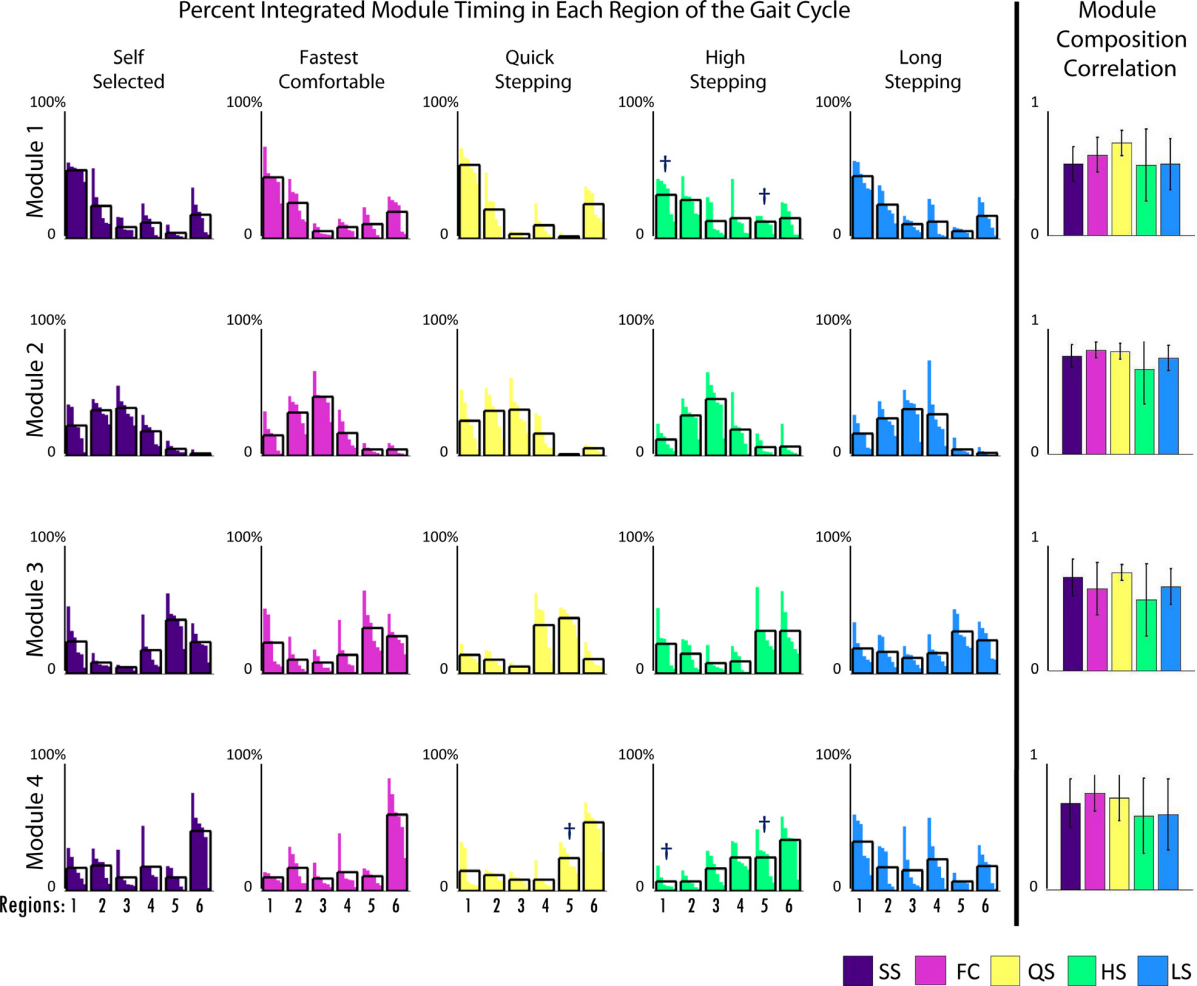
The first five bar graphs on the left represent the percent of total integrated module timing curve in each region of the gait cycle (Fig. 1) for poststroke subjects with four modules (*n *=**6). Analysis of variances (ANOVAs) were run for each module and region of the gait cycle across mobility capability tasks. Asterisks show significance (*α *< 0.05) and “†” shows marginal significance (*α *< 0.1) in post hoc *t*‐tests with Bonferroni corrections compared to the self‐selected (SS) condition only. The last column shows Pearson's correlations of module composition of poststroke subjects with four modules to average module composition of control subjects. ANOVAs were run for each module across mobility capability tasks. Asterisks show significance (*α *< 0.05) in post hoc *t*‐tests with Bonferroni corrections compared to the SS condition only.

The module compositions found in the four‐module poststroke subjects were similar to those of the control subjects (compare Fig. 5 to Fig. 3) such that the correlation with the average control modules was always >0.6 (Fig. 6). For the poststroke subjects with four modules, each module's composition did not differ between mobility capability tasks.

Module timings in the poststroke subjects with four modules were similar to the control subjects (Fig. 5). Despite some visual differences in the average curves between mobility tasks, the percent of integrated module timing in the six regions of the gait cycle were not significantly different between any of the tasks (Fig. 6). Specifically, the average timing curve of Module 4 did have two peaks during HS as it did for the control subjects (Fig. 5, last column – note similar shape as in Fig. 3), but there was a larger standard deviation poststroke than in the control subjects. Indeed, Module 4 in region 5 (early swing; *P* = 0.02) had significantly (*α *< 0.05 for post hoc *t*‐tests) less activity in LS than QS and HS, and marginally less (*α *= 0.0665 for post hoc *t*‐tests) activity in SS than QS and HS. Additionally, the average timing curve of Module 4 peaked late in swing during LS as it did for the control subjects (compare Figs. 3, 5), although there were no significant differences between LS and SS walking.

### Poststroke subjects with less than four modules

**Figure 7.**
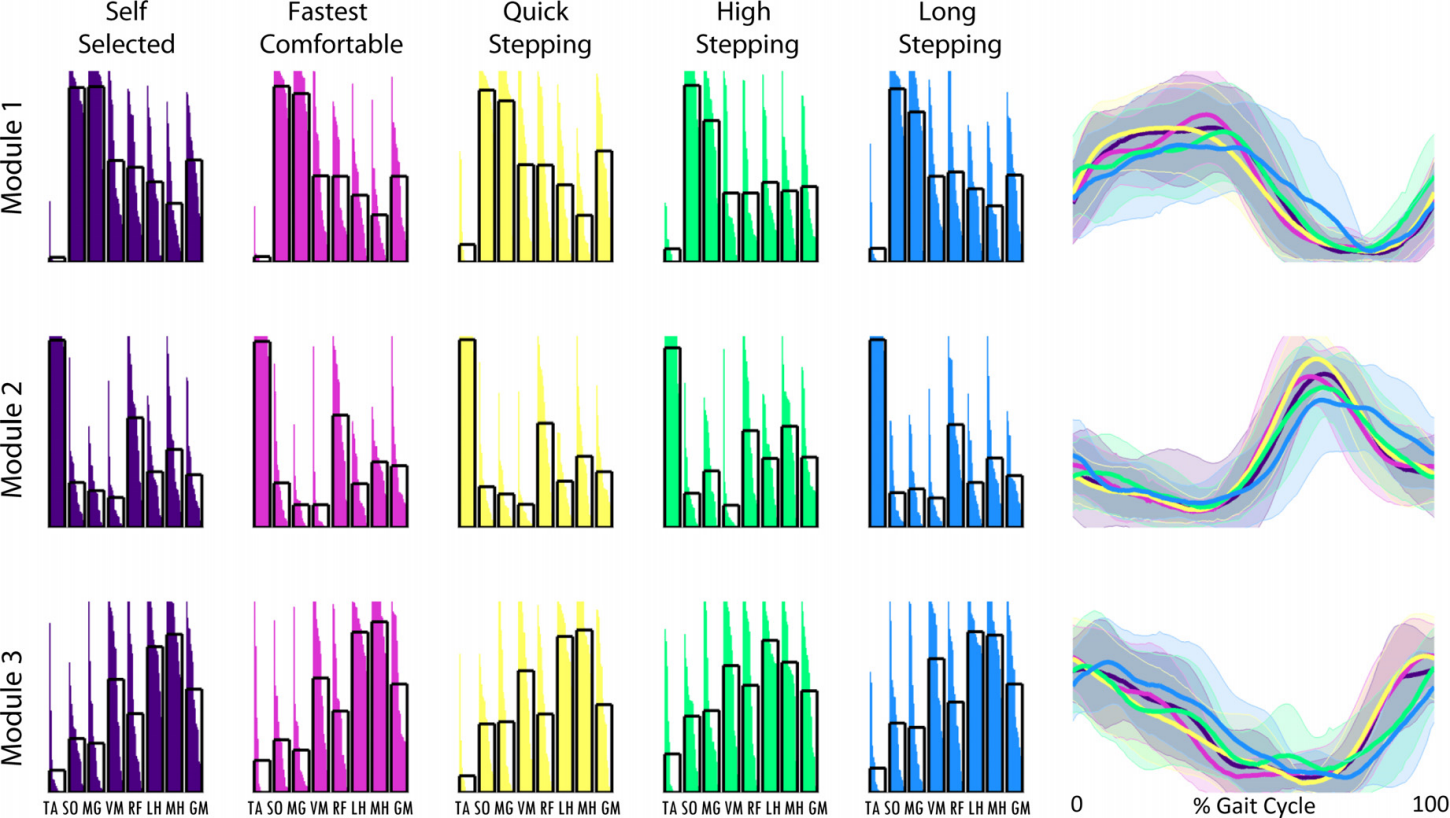
Average poststroke subject modules for subjects with three modules are shown for all tasks. Module compositions are on the left and timing of the correspondingly colored module are on the right.

**Figure 8.**
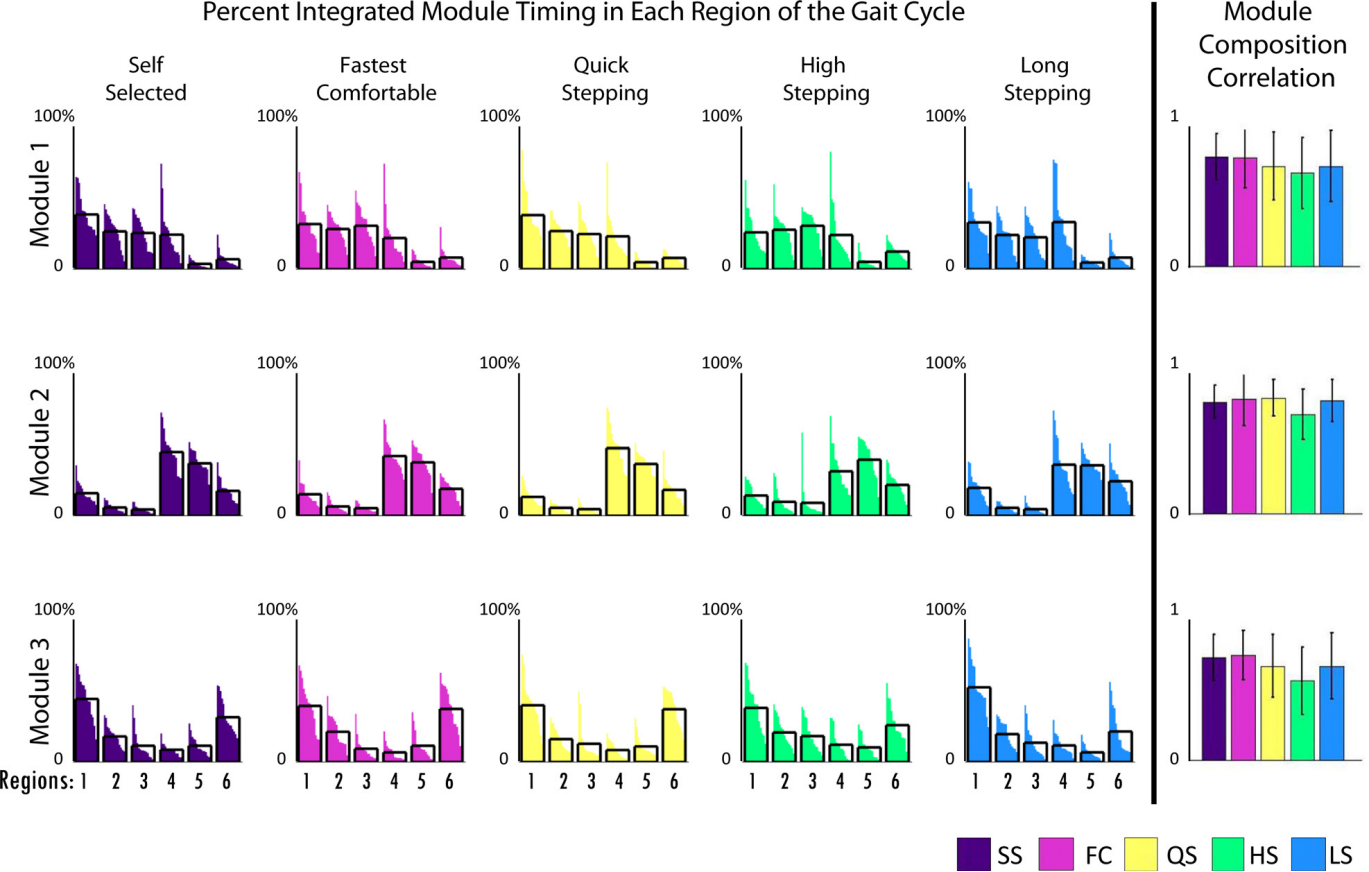
The first five bar graphs on the left represent the percent of total integrated module timing curve in each region of the gait cycle (Fig. 1) for post stroke subjects with three modules (*n *=**15). Analysis of variances (ANOVAs) were run for each module and region of the gait cycle across mobility capability tasks. Asterisks show significance (*α *< 0.05) and “†” shows marginal significance (*α *< 0.1) in post hoc *t*‐tests with Bonferroni corrections compared to the self‐selected (SS) condition only. The last column shows Pearson's correlations of module composition of poststroke subjects with three modules to average module composition of poststroke subjects with three modules. ANOVAs were run for each module across mobility capability tasks. Asterisks show significance (*α *< 0.05) in post hoc *t*‐tests with Bonferroni corrections compared to the SS condition only.

**Figure 9.**
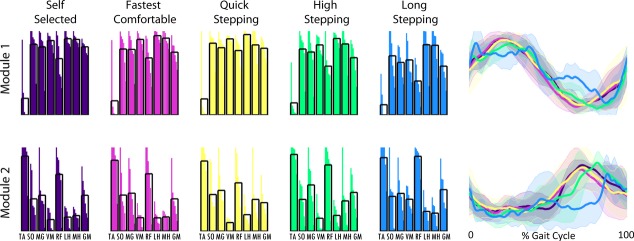
Average poststroke subject modules for subjects with two modules are shown for all tasks. Module compositions are on the left and timing of the correspondingly colored module are on the right.

**Figure 10.**
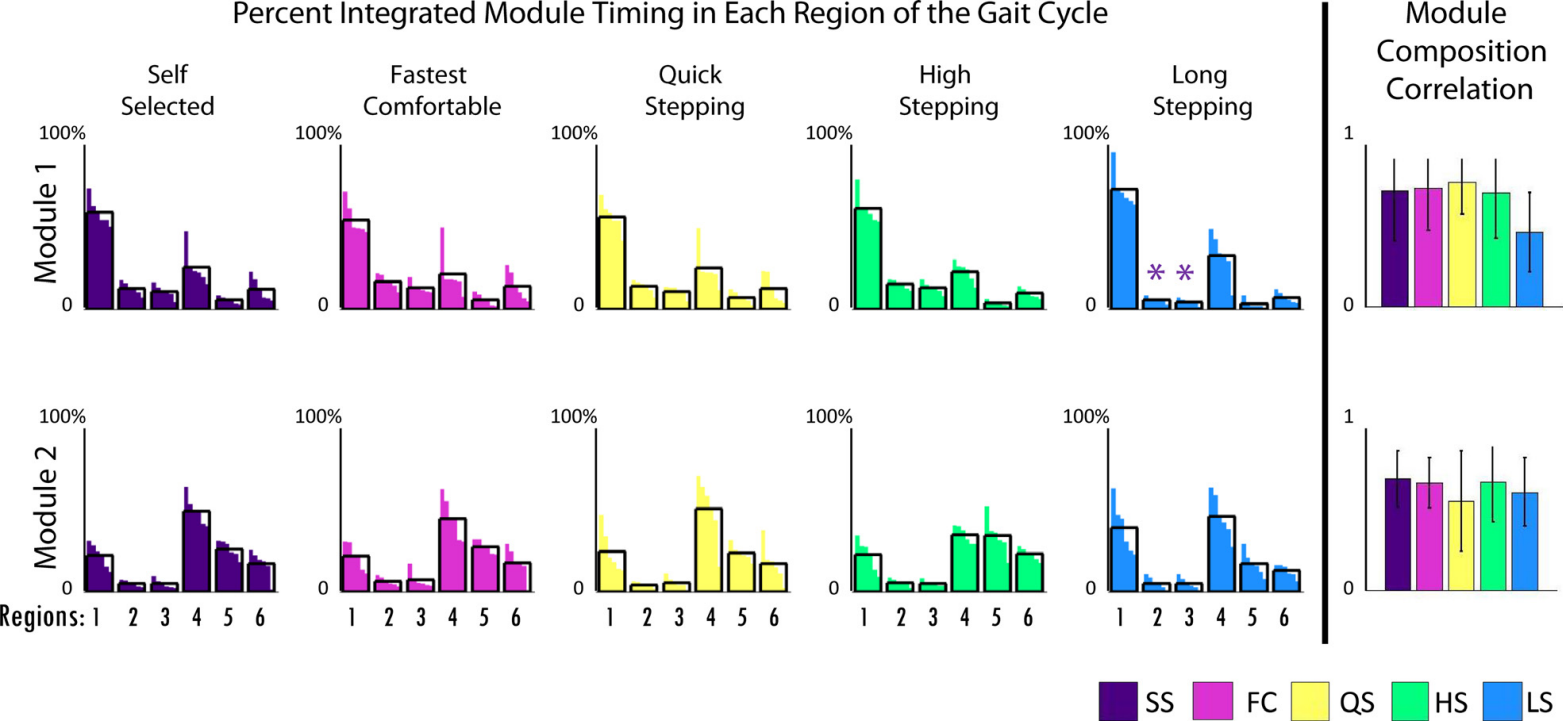
The first five bar graphs on the left represent the percent of total integrated module timing curve in each region of the gait cycle (Fig. 1) for poststroke subjects with two modules (*n *=**6). Analysis of variances (ANOVAs) were run for each module and region of the gait cycle across mobility capability tasks. Asterisks show significance (*α *< 0.05) and “†” shows marginal significance (*α *< 0.1) in post hoc *t*‐tests with Bonferroni corrections compared to the self‐selected (SS) condition only. The last column shows Pearson's correlations of module composition of poststroke subjects with two modules to average module composition of poststroke subjects with two modules. ANOVAs were run for each module across mobility capability tasks. Asterisks show significance (*α *< 0.05) in post hoc *t*‐tests with Bonferroni corrections compared to the SS condition only.

Poststroke subjects with less than four modules also maintained consistency in their composition across the mobility tasks (Figs. 7–10). Module timing was also not significantly different for any of the subjects with three modules across the mobility capability tasks. Module timing did change during LS for subjects with two modules (Fig. 10) with a decreased activation of their Module 1 during mid‐stance (regions 2 and 3) compared to SS walking. All of the other tasks in subjects with two modules had similar timing profiles to one another.

### Mobility capability

**Figure 11.**
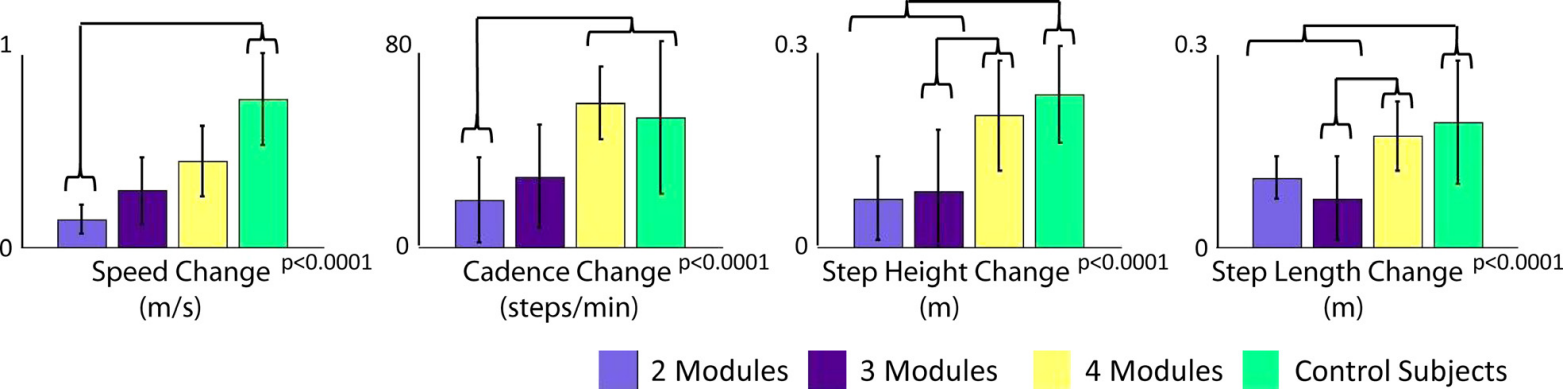
Mobility capability in each task by subject group. *P*‐values are indicated for one‐way analysis of variances (ANOVAs) across groups of subjects (e.g., poststroke subjects with two modules, three modules, four modules, and control subjects) for each mobility capability measure. Brackets indicate significance in post hoc *t*‐tests with Bonferroni corrections.

In addition to having lower correlations to the average control modules and more limited ability to change module timing, the poststroke subjects were not able to perform the mobility capability measures as well as the control subjects. The ability to change speed (*P* < 0.0001), cadence (*P* < 0.0001), step height (*P* < 0.0001), and step length (*P* < 0.0001) all corresponded to the number of modules in poststroke subjects and these abilities are reduced compared to the control subjects (Fig. 11).

## Discussion

The goal of this study was to determine whether the same modules would be used to perform a range of locomotor tasks, with each subject modifying the timing and magnitude of those modules to adapt to the new biomechanical demands of each task. Overall, we found that for each subject the same underlying modules (number and composition) that contribute to steady‐state walking also contribute to mobility capability tasks (e.g., FC, HS, LS, and QS) in healthy and poststroke subjects. Furthermore, we found that subjects with fewer modules performed the tasks more poorly. Thus, our theoretical framework was mostly supported. We expected that the same modules would be used to perform the range of locomotor tasks, with each subject modifying the timing and magnitude of those modules to adapt to the new biomechanical demands of each task. Furthermore, since we believe that the modules each result in the performance of different biomechanical functions, we expected that the lack of four independent modules with similar composition, timing, and magnitude would degrade performance of the locomotor tasks. Of particular interest, the lack of four independent modules showed up very strongly in the task performances of high stepping and long stepping, the tasks that showed the greatest changes in module timing in healthy subjects. It appears that three or two modules did not yield the adaptability of four modules and task performance suffered.

### Control subjects

In healthy subjects, module timings, but not compositions, changed when the functional task demands were altered. The compositions of the four modules for all mobility capability tasks in the control subjects were consistent with control modules previously identified during steady‐state treadmill walking (Clark et al. 2010; Routson et al. 2013) and the SS walking data collected in this study (Fig. 3). The only significant difference in module compositions occurred in Module 4 between the LS and QS mobility tasks. This was likely due to a higher contribution of the vastus medialis in some of the subjects to Module 4 during the LS mobility task (Fig. 3). However, the average contribution of the vastus medialis to Module 4 remained below the 0.4 threshold for being a major contributor to that module (Neptune et al. 2009; Allen and Neptune 2012) and neither LS nor QS module composition correlations were significantly different from the SS walking condition. In addition, all of the correlations to the SS walking composition averages were large (>0.65), indicating very little variation in module compositions between each mobility task and SS walking. It is possible that average correlations as low as 0.65 may not necessarily be interpreted as similarity. However, correlations for muscle weightings for dissimilar modules have been shown to be between 0.07 and 0.4 (Clark et al. 2010) and the range for good agreement in module similarity has been reported to start as low as 0.65 (Oliveira et al. 2013). These results are consistent with previous studies showing that module compositions remain unchanged across speeds in both running and walking (Cappellini et al. 2006; Clark et al. 2010). These findings suggest that module compositions are preserved across functional demands while walking and provide further evidence that a consistent set of neural building blocks may exist to perform a variety of human locomotor tasks.

Module timings, however, were affected by different functional demands, particularly in QS, HS, and LS walking. In QS walking, all four modules had increased activation compared to SS walking in the regions preceding peak activation. In HS compared to SS walking, Module 4 had increased activation in preswing and early swing. Additionally in HS, Module 3 was active throughout the duration of swing versus the short burst in mid‐swing during SS walking. In LS walking, Module 4 was activated later in swing and longer into stance than in SS walking. Adaptability in module timings is consistent with previous studies that show the same modules found in steady‐state walking are also present in running and cutting maneuvers, but there exist differences in module timings (Cappellini et al. 2006; Oliveira et al. 2013).

### Poststroke subjects with four modules

In addition to having reduced correlations with the average control module compositions, indicating poorer module quality (Routson et al. 2013), the poststroke subjects with four modules also demonstrated less adaptability in module timing with changing functional demands. In control subjects during HS, there was increased Module 4 activity that occurred in preswing and early swing. However, in poststroke subjects, the Module 4 activity in preswing and early swing was not always present. Thus, there was a higher standard deviation in the average module timing and no significance in the comparison of percent integrated module timing for those regions compared to SS walking. This finding is consistent with previous studies showing that even though subjects may have four modules poststroke, those modules can result in poorer walking performance and can differ in composition and timing from those in healthy subjects (Clark et al. 2010; Routson et al. 2013).

### Poststroke subjects with less than four modules

Subjects with less than four modules poststroke also had consistent module compositions across mobility tasks. Subjects with three modules poststroke had no adaptability in module timing with the mobility tasks. In contrast, subjects with two modules demonstrated differences in the timing of their Module 1 (all muscles except TA and RF and consistent with merged Modules 1, 2, and 4 of the control subjects) in LS compared to SS walking. This decrease is not a key finding as the mid‐stance region already has very little activity for that module in SS walking. Note that there was a sharp drop in performance from four to three modules in QS, HS, and LS, the tasks that control subjects showed the greatest changes in module timing. Not having the four independent modules appears to greatly affect performance. Previous studies have shown that in subjects with less than four modules, the merging of modules interferes with the successful execution of specific biomechanical functions (Clark et al. 2010; Allen et al. 2013). This study suggests that the merging of modules may also adversely affect the ability to adapt timings in order to execute task‐specific goals.

### Mobility capability

The number of modules poststroke not only affects walking performance (Clark et al. 2010; Allen et al. 2013; Routson et al. 2013) but also mobility capability (Fig. 11). Subjects with two modules poststroke demonstrated significantly less change in speed, cadence, step height, and step length than control subjects and significantly less cadence change than poststroke subjects with four modules. Subjects with three modules had significantly less change in step height and step length than control subjects and poststroke subjects with four modules. This suggests that in subjects poststroke, the number of modules is indicative of not only typical walking performance but also of mobility capability performance. Since the number of modules can be increased with locomotor therapy, which improves gait performance (Routson et al. 2013), it is also likely that mobility capability can also be influenced by rehabilitative therapy.

For the tasks investigated in this study, adaptability of the timing of Module 4 (hamstrings) appears particularly important; however, it was not modified in poststroke subjects. Hamstring weakness and temporal irregularity are common in hemiparetic gait (Den Otter et al. 2007; Routson et al. 2013). In healthy SS walking, Module 4 contributes to forward propulsion and accelerates the body laterally during the first half of stance and decelerates the ipsilateral leg in late swing (Neptune et al. 2009; Allen and Neptune 2012). Therefore, it is likely that Module 4 weakness and poor timing adversely affect mobility. Indeed, a recent simulation analysis showed that when timing of Module 4 is altered, body support, forward propulsion, and leg swing are all adversely affected (Allen et al. 2013). Our study's findings further suggest that the ability to adapt the timing of Module 4 during HS and LS tasks influences the mobility capability performance.

### Methodological considerations

The existence and function of muscle modules is still currently disputed (Tresch and Jarc 2009). We and others interpret muscle modules as fixed coexcited groups of muscles that contribute toward specific biomechanical function (Ting and Macpherson 2005; Clark et al. 2010). However, this interpretation is not universal. Some believe that modules may develop due to optimal control (de Rugy et al. 2013) or emerge as the result of biomechanical constraints (Kutch and Valero‐Cuevas 2012). Recent studies have provided evidence against the existence of muscle modules in finger control (Kutch et al. 2008; Valero‐Cuevas et al. 2009). Yet, the lack of modules found in the finger does not definitively prove that modules do not exist in all limbs and for all mobility tasks. For example, modules have still been used to explain movements of the arm (Krishnamoorthy et al. 2007) and hand (Gentner and Classen 2006; Ajiboye and Weir 2009). Therefore, it is possible that nonspecialized and repetitive movements may still be governed by modules. Furthermore, several simulation studies have shown the ability of a limited number of modules to produce realistic and well‐coordinated locomotion (e.g., Neptune et al. 2009; Allen and Neptune 2012). This provides promising evidence that successful walking may be the product of ongoing modulation of the excitation modules based on task objectives and feedback of the system state. Modules have also been observed in neonates (Dominici et al. 2011) in addition to a wide range of locomotor activities in adults such as walking (Ivanenko et al. 2004; Clark et al. 2010), running (Cappellini et al. 2006), cutting maneuvers (Oliveira et al. 2013), cycling (Hug et al. 2010), and postural responses (Torres‐Oviedo and Ting 2007). It is possible that task and biomechanical constraints could reduce the redundancy in the system and restrict the set of muscle activation patterns observed during human locomotion. Thus, our tasks may not be different enough from one another to significantly affect module number and composition. Future work should be directed at examining additional locomotor tasks and perhaps more direct measures of neural activations to provide a definitive neural basis for the existence of modules.

In addition, it is important to note that the focus of our research into modules in hemiparetic motor control in this and previous studies (Clark et al. 2010; Allen et al. 2013; Routson et al. 2013) has been on understanding whether muscle activity exhibits independence in timing from the mass flexion and extension patterns commonly seen clinically because we wish to understand the biomechanical consequences of abnormal muscle coactivation (represented by present, absent, or merged modules). Consistent with this focus, the validity of our results are not exclusively dependent on whether modules are fixed in composition or not.

Due to our limited recording of EMG from eight muscles, we were only able to identify four modules during healthy control walking. Recent studies have shown that five to six modules are necessary to control healthy walking (Ivanenko et al. 2004; Neptune et al. 2009; Allen and Neptune 2012). However, the analysis revealed similar modules like those previously identified in stroke subjects with this same set of muscles (Clark et al. 2010; Routson et al. 2013). In addition, recent simulation work has shown that the number and choice of muscles may impact number and composition of muscle modules identified (Steele et al. 2013). However, because it is not possible to collect reliable surface EMG from the majority of the lower limb muscles of stroke patients, we focused on the main contributors to biomechanical subtasks of walking. We believe that the number of muscles we collected EMG from is sufficient for the scope of this study because of the way that we and others have interpreted modular analysis such that fixed groups of muscles are coexcited to perform specific biomechanical functions (Tresch et al. 1999; Ting and Macpherson 2005; Clark et al. 2010). In addition, a recent study analyzing modules in multidirectional human locomotion (forward, backward, and sideways walking) collected EMG from 25 muscles and found that five to seven (an average of 5.8 ± 0.7) modules were sufficient to reconstruct EMG for each task with their individual‐muscle evaluation criteria. Furthermore, four to six modules were sufficient using a grouped‐muscle criteria and consistent with previous studies (Zelik et al. 2014). This suggests that more muscles are not essential to performing a modular analysis, as long as EMG is collected from the main contributors to the locomotor activity. Finally, our previous simulation work was able to confirm that the four experimentally identified modules, in addition to two bimodal modules, were able to produce well‐coordinated walking simulations of both healthy and poststroke subjects (Neptune et al. 2009; Allen and Neptune 2012; Allen et al. 2013). Thus, we are confident that the muscles from which we collected EMG were the most appropriate for our analyses. Future work will include musculoskeletal modeling and simulation studies of this data to develop a more complete understanding of the modules needed to perform specific mobility capability tasks.

In addition to modular pattern dissimilarity, which reflects altered neural control, muscle weakness may also play a role in the successful execution of the mobility capability tasks. Our methods did not include an analysis to determine whether a particular subject's muscle strength was sufficient to perform the mobility capability tasks. However, this study does provide evidence that module number and composition are associated with successful mobility task performance.

Additionally, by using the number of modules found for SS walking in each subject for all of their mobility capability tasks, we may have overlooked the ability of some subjects to change the number of modules they use for a particular task. However, a one‐way ANOVA was run for *all* subjects across mobility tasks and there were no significant differences in the number of modules for any particular task.

## Conclusion

In conclusion, we found that although some poststroke subjects had a smaller number of modules than healthy subjects, the same underlying modules (number and composition) in each subject (both healthy and poststroke) that contribute to SS walking also contribute to specific mobility capability tasks (i.e., FC, HS, LS, and QS) in those subjects. In healthy subjects, we found that module timing, but not composition, changes when functional task demands are altered during walking. However, this adaptability in module timing is limited in poststroke subjects, which limits their mobility capability performance. In addition, the greater number of modules poststroke indicates superior mobility capability. Thus, we found specific tasks required greater changes in module timing and were then performed more poorly by subjects who did not have all of the independent modules. These specific tasks may provide a basis for a clinical assessment that reveals information about the status of the underlying health of neural (modular) organization. Thus, therapies targeting improved module timing adaptability during these tasks in addition to the separation of merged modules may lead to enhanced mobility capability in persons poststroke.

## Acknowledgments

The contents are solely the responsibility of the authors and do not necessarily represent the official views of the National Institutes of Health, NICHD, National Science Foundation, the Department of Veterans Affairs or the United States Government.

## Conflict of Interest

None declared.
